# Carbapenem-Resistant and Extended-Spectrum β-Lactamase–Producing Enterobacterales in Children, United States, 2016–2020

**DOI:** 10.3201/eid3006.231734

**Published:** 2024-06

**Authors:** Heather N. Grome, Julian E. Grass, Nadezhda Duffy, Sandra N. Bulens, Uzma Ansari, Davina Campbell, Joseph D. Lutgring, Amy S. Gargis, Thao Masters, Alyssa G. Kent, Susannah L. McKay, Gillian Smith, Lucy E. Wilson, Elisabeth Vaeth, Bailey Evenson, Ghinwa Dumyati, Rebecca Tsay, Erin Phipps, Kristina Flores, Christopher D. Wilson, Christopher A. Czaja, Helen Johnston, Sarah J. Janelle, Ruth Lynfield, Sean O’Malley, Paula Snippes Vagnone, Meghan Maloney, Joelle Nadle, Alice Y. Guh

**Affiliations:** Centers for Disease Control and Prevention, Atlanta, Georgia, USA (H.N. Grome, J.E. Grass, N. Duffy, S.N. Bulens, U. Ansari, D. Campbell, J.D. Lutgring, A.S. Gargis, T. Masters, A.G. Kent, S.L. McKay, A.Y. Guh);; Foundation for Atlanta Veterans Education and Research, Decatur, Georgia, USA (G. Smith);; Atlanta Veterans Affairs Medical Center, Decatur (G. Smith);; Maryland Department of Health, Baltimore, Maryland, USA (L.E. Wilson, E. Vaeth, B. Evenson);; New York Rochester Emerging Infections Program at the University of Rochester Medical Center, Rochester, New York, USA (G. Dumyati, R. Tsay);; University of New Mexico, Albuquerque, New Mexico, USA (E. Phipps, K. Flores);; New Mexico Emerging Infections Program, Santa Fe, New Mexico, USA (E. Phipps, K. Flores);; Tennessee Department of Health, Nashville, Tennessee, USA (C.D. Wilson);; Colorado Department of Public Health and Environment, Denver, Colorado, USA (C.A. Czaja, H. Johnston, S.J. Janelle);; Minnesota Department of Health, St. Paul, Minnesota, USA (R. Lynfield, S. O’Malley, P. Snippes Vagnone);; Connecticut Department of Public Health, Hartford, Connecticut, USA (M. Maloney);; California Emerging Infections Program, Oakland, California, USA (J. Nadle)

**Keywords:** Enterobacterales, carbapenem-resistant Enterobacterales, extended-spectrum β-lactamase-producing Enterobacterales, antimicrobial resistance, epidemiology, child, public health, communicable diseases, bacteria, United States

## Abstract

2019–2020 at 6 US sites. Among 159 CRE cases in children (median age 5 years), CRE was isolated from urine for 131 (82.4%) and blood from 20 (12.6%). Annual CRE incidence rate (cases/100,000 population) was 0.47–0.87. Among 207 ESBL-E cases in children (median age 6 years), ESBL-E was isolated from urine of 196 (94.7%) and blood of 8 (3.9%). Annual ESBL-E incidence rate was 26.5 in 2019 and 19.63 in 2020. CRE and ESBL-E rates were >2-fold higher among infants than other age groups. Most CRE and ESBL-E cases were healthcare-associated community-onset (68 [43.0%] for CRE vs. 40 [23.7%] for ESBL-E) or community-associated (43 [27.2%] for CRE vs. 109 [64.5%] for ESBL-E). Programs to detect, prevent, and treat multidrug-resistant infections must include pediatric populations (particularly the youngest) and outpatient settings.

Increasing antimicrobial resistance (AMR) remains a critical public health threat (*1*,*2*). Carbapenem-resistant Enterobacterales (CRE) have been identified as an urgent public health threat and extended-spectrum β-lactamase (ESBL)–producing Enterobacterales (ESBL-E) as a serious public health threat (*1*). Both bacteria types remain of concern because of transmissibility of the AMR genes they harbor and limited treatment options. Particularly concerning are plasmid-mediated resistance mechanisms in which genes encoding carbapenemases and ESBLs, as well as other resistance determinants, can disseminate between different organisms, thus furthering the spread of CRE and ESBL-E (*3*). Knowledge of the burden of these infections has implications for public health and the control strategies needed to prevent spread in adult and pediatric populations.

Most US studies have focused on risk factors for infection or colonization of CRE and ESBL-E in adults; national epidemiologic data for children are comparatively lacking (*4*–*9*). Moreover, attention to antimicrobial-resistant bacterial infections in children has perhaps been further disrupted by the COVID-19 pandemic, and trends have potentially worsened over the past 3 years. Few antimicrobial drugs can treat CRE and ESBL-E infections (*10*), and limited pediatric-specific clinical trials of antimicrobial drugs contribute to a scarcity of knowledge with regard to children compared with adults (*11*). A small number of studies have described the continued emergence of AMR mechanisms in US children (*12*–*16*) and identified variations in epidemiology by hospital and bacteria species across multiple pediatric medical centers (*17*–*19*). However, most studies were conducted in earlier years and were not designed to characterize the clinical and molecular features of cases identified from community and hospital settings on a population level.

The Centers for Disease Control and Prevention (CDC) Emerging Infections Program (EIP) conducts laboratory and population-based surveillance for CRE and ESBL-E in diverse US sites through the Multi-site Gram-negative Surveillance Initiative (https://www.cdc.gov/hai/eip/mugsi.html). Using those data, we focused on the descriptive and comparative epidemiology of CRE and ESBL-E, 2 of the most pressing gram-negative bacteria resistance threats, in US children.

Our study activity was reviewed by CDC, deemed not research, and was conducted consistent with applicable federal law and CDC policy (e.g., 45 C.F.R. part 46.102(l)(2), 21 C.F.R. part 56; 42 U.S.C. §241(d); 5 U.S.C. §552a; 44 U.S.C. §3501 et seq.). Similarly, the protocol was reviewed by all participating EIP sites and either was deemed nonresearch or received institutional review board approval with a waiver of informed consent.

## Methods

### Surveillance Population

As of 2016, county-level CRE surveillance was conducted in selected US metropolitan counties at 8 EIP sites (Colorado, Georgia, Maryland, Minnesota, New Mexico, New York, Oregon, Tennessee); surveillance subsequently expanded to 2 additional sites (California in 2017 and Connecticut statewide in 2018) (*20*). The total population of the 10 participating areas under surveillance in 2020 was an estimated 23.2 million, of which an estimated 4.9 million were children (*21*).

County-level ESBL-E surveillance started in July 2019 in selected counties at 6 EIP sites (Colorado, Georgia, Maryland, New Mexico, New York, Tennessee). The total population of the 6 participating areas under surveillance in 2020 was an estimated 3.0 million, of which an estimated 626,000 were children (*21*) (Appendix).

### Case Definitions and Data Collection

Beginning in 2016, we defined an incident pediatric CRE case as the first isolation during a 30-day period of *Klebsiella pneumoniae*, *K. oxytoca*, *K. aerogenes*, *Enterobacter cloacae* complex, or *Escherichia coli* resistant to >1 carbapenem (imipenem, meropenem, doripenem, ertapenem) from a normally sterile body site (Appendix) or urine specimen from a surveillance area resident <18 years of age. We defined an incident pediatric ESBL-E case as the first isolation during a 30-day period of *E. coli*, *K. pneumoniae*, *K. variicola*, or *K. oxytoca* resistant to >1 extended-spectrum cephalosporin (ceftazidime, cefotaxime, or ceftriaxone) and nonresistant (i.e., susceptible or intermediate) to all tested carbapenems from a normally sterile body site or urine specimen from a surveillance area resident <18 years of age. To prevent duplication with CRE surveillance, we excluded ESBL-E isolates that were carbapenem resistant. For both ESBL-E and CRE, if a new specimen meeting the case definition was collected >30 days after the patient’s last incident case with the same organism, it was also reported as an incident case. CRE and ESBL-E cases were identified through a query of automated testing instruments based on laboratory protocols (Appendix).

All incident CRE cases, as well as all incident ESBL-E cases from a sterile source, underwent medical record review by using a standardized case report form to collect patient demographics, underlying conditions, healthcare exposures and outcomes, location of specimen collection, associated infection types, and antimicrobial susceptibility testing results (*20*) (Appendix). For ESBL-E cases identified from urine sources, for each year, first incident cases per species in a patient were reviewed.

Cases were considered hospital onset if the incident culture was collected >3 days after hospital admission. All other cases were considered community onset and further classified as either 1) healthcare-associated if the person had hospitalization, surgery, residence in a long-term care facility or long-term care acute care hospital, or chronic dialysis in the year before culture or had an indwelling device in the 2 days before culture; or 2) community-associated if none of those risk factors were identified.

### Isolate Collection

We submitted a convenience sample of isolates from all EIP sites to CDC for confirmatory and molecular characterization. CRE and ESBL-E isolates underwent species identification by matrix-assisted laser desorption/ionization time-of-flight mass spectrometry (MALDI-TOF Biotyper 3.1; Bruker Daltronics, https://www.bruker.com). We conducted antimicrobial susceptibility testing of CRE isolates by using reference broth microdilution with a metallo-β-lactamase screen (*22*,*23*), screening for carbapenemases by using the modified carbapenem inactivation method (*24*), and real-time PCR testing for *bla*_KPC_, *bla*_NDM_, *bla*_VIM_, *bla*_IMP_, and *bla*_OXA-48-like_ genes (*25*–*28*). If a CRE isolate harbored a carbapenemase-producing gene according to PCR, it was classified as carbapenemase producing (CP). We used real-time PCR to screen all isolates with a colistin MIC >2 µg/mL for plasmid-mediated colistin resistance genes (*mcr*-1 and *mcr*-2) (*29*). We conducted antimicrobial susceptibility testing of ESBL-E by using reference broth microdilution and performed phenotypic screening for ESBL production with ceftazidime and cefotaxime alone and in combination with clavulanate (*24*). We conducted whole-genome sequencing on a subset of CRE isolates from 2016–2018 that were confirmed to be carbapenem resistant and on the subset of ESBL isolates received from CDC (Appendix).

### Statistical Analyses

We calculated crude incidence rates by using case counts and 2016–2020 US Census estimates of the surveillance area population <18 years of age. For incidence rates presented by region, demographics, or age group, denominators represent the pediatric population also stratified by that subgroup. Analysis was limited to case report forms completed as of November 29, 2022. We performed descriptive and comparative analyses for CRE and ESBL-E cases by using the χ^2^ test or the Fisher exact test (where applicable) for categorical variables and the Wilcoxon rank sum test for continuous variables. We used SAS version 9.4 (SAS Institute Inc, https://www.sas.com) to conduct data analyses.

## Results

### Cases and Incidence Rates

During 2016–2020, a total of 159 incident CRE cases were identified in 142 children across 10 EIP sites. Of the 159 cases, 83 (52.2%) isolates were *E. cloacae* complex, 50 (31.5%) *E. coli*, 17 (10.7%) *K. pneumoniae*, 5 (3.1%) *K. aerogenes*, and 4 (2.5%) *K. oxytoca* (Table 1). The number of CRE cases per EIP site ranged from 3 to 47. *E. coli* comprised half or most of the CRE cases in New Mexico (50.0%), Tennessee (71.4%), and California (85.7%), whereas *E. cloacae* complex were the most common organisms at the other sites (44.4%–71.4%). Of the 142 unique persons with CRE, during the 5-year surveillance period >2 incident cultures were obtained from 17 (12.0%) (range 2–6 episodes).

**Table 1:** Incident CP and ESBL-E–producing Enterobacterales cases in children, by organism, United States*

Organism	Incident CRE cases, 2016–2020							Incident ESBL-E cases, 2019–2020†		
	No. (%) cases	Isolates submitted for carbapenemase testing, no.	No. (%) CP isolates‡	No. (%) carbapenemase genes‡				No. (%) cases	No. isolates submitted for ESBL testing	No. (%) ESBL-producing organisms§
				*bla*_KPC_	*bla*_NDM_	*bla*_OXA-48_-like				
*Escherichia coli*	50 (31.5)	26	5 (19.2)	0	2 (7.7)	3 (11.5)		182 (87.9)	16	15 (93.8)
*Enterobacter cloacae c*omplex	83 (52.2)	47	1 (2.1)	1 (2.1)	0	0		NA	NA	NA
*Klebsiella aerogenes*	5 (3.1)	4	1 (25.5)	0	1 (25.0)	0		NA	NA	NA
*K. oxytoca*	4 (2.5)	1	1 (100.0)	1 (100)	0	0		2 (1.0)	NA	NA
*K. pneumoniae*	17 (10.7)	8	1 (12.5)	0	1 (12.5)	0		23 (11.1)	3	3 (100.0)
Total	159	86	9 (10.5)	2 (2.3)	4 (4.7)	3 (3.5)		207	19	18 (94.7)

*All incident pediatric cases with available case report form data as of November 28, 2022, were included in this analysis. CP, carbapenemase-producing; CRE, carbapenem-resistant Enterobacterales; ESBL-E, extended-spectrum β-lactamase–producing Enterobacterales; NA, not applicable (no organisms tested; ESBL-E surveillance included *Escherichia coli, Klebsiella oxytoca, Klebsiella pneumoniae*). †ESBL-E surveillance began in July 2019 at all participating sites. California, Connecticut, Minnesota, and Oregon do not participate in ESBL-E surveillance. ‡Percentages shown are of isolates submitted. Carbapenemases and colistin resistance genes not listed in the table were not detected for any isolates. §Percentages shown are of isolates submitted. Phenotypic screening for ESBL-E production was performed by using ceftazidime and cefotaxime alone and in combination with clavulanate according to Clinical and Laboratory Standards Institute guidelines (*24*).

During 2019–2020, a total of 207 incident ESBL-E cases were identified in 184 children across the 6 participating EIP sites. Of the 207 cases, 182 (87.9%) isolates were *E. coli*, 23 (11.1%) *K. pneumoniae*, and 2 (1.0%) *K. oxytoca* (Table 1). The number of ESBL-E cases per EIP site ranged from 14 to 57 cases; at all 6 sites, the predominant organism was *E. coli* (82.5%–100.0%). Of the 184 unique persons with ESBL-E, during the 1.5-year surveillance period, >2 incident cultures were obtained from 23 (12.5%) (range 2–4 episodes).

The overall annual CRE incidence rate (cases/100,000 pediatric population) across EIP sites during the 5-year period was 0.70 (range 0.47–0.87). The overall annual ESBL-E incidence rate during the 1.5-year period was 23.08, decreasing from 26.54 in 2019 to 19.63 in 2020. Crude incidence rates for CRE and ESBL-E varied by geographic region and year (Table 2).

**Table 2:** Incident pediatric CRE and ESBL-E cases with annual crude incidence, by geographic regions and demographic characteristics, United States*

Category	Crude annual incidence rate							
	Incident CRE cases						Incident ESBL-E cases	
2016	2017	2018	2019	2020	2019‡	2020
EIP sites by geographic region†								
Northeast	0.63	0.00	1.24	0.91	1.38		26.15	24.39
Midwest	1.25	0.74	0.25	0.99	0.99		NA	NA
South	0.82	0.55	0.71	1.05	0.44		26.30	16.57
West	0.86	0.37	0.48	0.64	0.38		27.15	20.15
Total	0.87	0.47	0.68	0.87	0.63		26.54	19.63
Demographic characteristics								
Sex§								
M	0.44	0.32	0.75	0.67	0.52		13.04	6.28
F	1.26	0.62	0.61	1.07	0.74		39.89	33.43
Race								
White	0.68	0.31	0.55	0.78	0.50		23.85	13.96
Black	0.57	0.32	0.57	1.05	0.38		12.79	5.92
Other¶	0.41	0.26	0.35	0.24	0.56		20.26	12.29

*Crude annual incidence rate, cases/100,000 pediatric population. CRE, carbapenem-resistant Enterobacterales; EIP, Emerging Infections Program; ESBL-E, extended-spectrum β-lactamase–producing Enterobacterales; NA, not applicable. †EIP sites grouped according to the 4 geographic regions defined by the US Census Bureau because of small case counts at some sites. California did not participate in CRE surveillance in 2016. Connecticut did not participate in CRE surveillance in 2016 or 2017. California, Connecticut, Minnesota, and Oregon do not participate in ESBL-E surveillance. ‡ESBL-E surveillance was completed for 6 months during 2019. Crude annual incidence rates estimated as the number of cases multiplied by 2 divided by population based on 2019 US Census for that year. §One CRE and 1 ESBL-E case had sex reported as unknown. ¶Other represents all reported races not indicated as White or Black, including American Indian or Alaska Native, Asian, Native Hawaiian or other Pacific Islander, or some other race. This combination category was necessary because of small numbers of pediatric cases in persons of races other than White or Black. In addition, 37 CRE cases and 74 ESBL-E cases had race indicated as unknown (not included in this category).

During 2016–2020, annual incidence rates for infants (children <1 year of age) were consistently higher than those for other age groups, ranging from 1.95 to 3.82 cases/100,000 pediatric population for CRE and 46.85 to 91.97 cases/100,000 pediatric population for ESBL-E (Figure). In addition, crude annual incidence rates for CRE and ESBL-E were nearly always higher for female than male children **(**Table 2), except in the youngest age group. During 2016–2020, average annual crude incidence rates for CRE cases were higher for male than for female children <1 year of age (3.38 vs. 2.35 cases/100,000 pediatric population).

**Figure:**
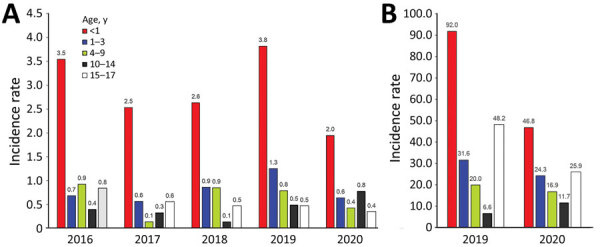
Annual crude incidence rates (cases/100,000 pediatric population) for incident pediatric carbapenem-resistant Enterobacterales (A) and extended-spectrum β-lactamase–producing Enterobacterales (B) cases, by age group, United States, 2016–2020. Incidence rate denominators are also stratified by age group.

### Demographics and Clinical Characteristics

Of 159 CRE cases, 94 (59.1%) were in girls (Table 3), compared with 49.1% of the overall pediatric population. Median age was 5 (interquartile range [IQR] 1–10) years; 4 (2.5%) children were <1 month of age and 31 (19.5%) were 1–12 months of age (compared with 5.4% of the population <1 year of age). Most children with CRE were White (79 [50.0%]) and non-Hispanic (86 [54.1%]). Similarly, of 207 ESBL-E cases, most were in girls (165 [79.7%] compared with 49.1% of the overall pediatric population), White (92 [44.4%]), and non-Hispanic (96 [46.4%]). The median age was 6 (IQR 2–15) years; 3 (1.5%) children were <1 month of age, and 27 (13.0%) were 1–12 months of age (compared with 5.1% of the population <1 year of age).

**Table 3:** Demographic and clinical characteristics of cases with CRE and ESBL-E infections, United States*

Characteristic	CRE, 2016–2020	ESBL-E, 2019–2020†	p value
Demographic	n = 159	n = 207	
Sex			**<0.01**
F	94 (59.1)	165 (79.7)	
M	64 (40.3)	41 (19.8)	
Unknown	1 (0.6)	1 (0.5)	
Age group, y			**<0.01**
Median, IQR	5 (1–10)	6 (2–15)	
<1	35 (22.0)	30 (14.5)	
1–3	30 (18.9)	40 (19.3)	
4–9	47 (29.6)	56 (27.1)	
10–14	28 (17.6)	27 (13.0)	
15–17	19 (12.0)	54 (26.1)	
Race			0.62
White	79 (50.0)	92 (44.4)	
Black	29 (18.2)	25 (12.1)	
Other	14 (8.8)	16 (7.7)	
Unknown	37 (23.3)	74 (35.8)	
Ethnicity			0.89
Hispanic	43 (27.0)	45 (21.7)	
Non-Hispanic	86 (54.1)	96 (46.4)	
Unknown	30 (18.9)	66 (31.9)	
Clinical‡			
Underlying conditions§	n = 158	n = 169	
Premature birth	20 (12.7)	11 (6.5)	0.06
Diabetes mellitus	0	2 (1.2)	0.27
Neurologic condition, any	34 (21.5)	20 (11.8)	**0.02**
Urinary tract problems/abnormalities	42 (26.6)	22 (13.0)	**<0.01**
Cardiovascular disease	13 (8.2)	3 (1.8)	**<0.01**
Chronic pulmonary disease	25 (15.8)	21 (12.4)	0.38
Chronic renal disease	24 (15.2)	3 (1.8)	**<0.01**
Gastrointestinal disease	3 (1.9)	1 (0.6)	0.23
Skin condition	12 (7.6)	8 (4.3)	0.28
Malignancy (hematologic or solid organ)	10 (6.3)	2 (1.2)	**<0.01**
Transplant (hematopoietic stem cell or solid organ)	15 (9.5)	1 (0.6)	**<0.01**
None	59 (37.3)	110 (65.1)	**<0.01**
Any condition	99 (62.7)	59 (34.9)	**<0.01**
Epidemiologic classification of incident cases			
Hospital onset	40 (25.3)	13 (7.7)	**<0.01**
Community-associated	43 (27.2)	109 (64.5)	**<0.01**
Healthcare-associated community onset	68 (43.0)	40 (23.7)	**<0.01**
Unknown	7 (4.4)	7 (4.1)	0.90

*Values are no. (%) except as indicated. Fisher exact test used for comparative statistics when >20% of expected cell counts were <5. Boldface indicates p<0.05. CRE, carbapenem-resistant Enterobacterales; ESBL-E, extended-spectrum β-lactamase–producing Enterobacterales. †ESBL-E surveillance was completed for 6 months during 2019. ‡Clinical characteristics were available for cases with completed case report forms only. §Cases could have >1 underlying condition associated with culture. Underlying conditions are further defined as follows: premature birth, birth before the week 37 of pregnancy, selected if medical record indicated premature birth and patient was <2 y of age; diabetes mellitus, includes both type I and type II; neurologic condition, any, includes cerebral palsy, chronic cognitive deficits, epilepsy/seizure/seizure disorders, multiple sclerosis, neuropathy, and others; urinary tract problems/abnormalities, a structural or functional urinary tract abnormality leading to obstruction or retention of urine as documented in the medical record; cardiovascular disease, includes congenital heart disease, congestive heart failure, prior cerebrovascular accident/stroke, peripheral vascular disease; chronic pulmonary disease, includes cystic fibrosis and any chronic respiratory condition resulting in chronic symptomatic dyspnea in medical record; chronic renal disease, includes chronic kidney disease (all stages), end-stage renal disease with or without dialysis; gastrointestinal disease, includes inflammatory bowel disease, liver disease, peptic ulcer disease, and short gut syndrome; skin condition, includes pressure ulcers, surgical wounds, other skin conditions such as eczema, psoriasis; malignancy, includes hematologic, metastatic and nonmetastatic solid organ; transplant, includes hematopoietic stem cell and solid organ.

Clinical characteristics were available for 158 CRE and 169 ESBL-E cases with completed case report forms (Table 3). Of those, a greater proportion of children with CRE than ESBL-E had a history of premature birth (20 [12.7%] vs. 11 [6.5%] among those with term birth; p = 0.06) and any underlying condition (99 [62.7%] vs. 59 [34.9%]; p<0.01).

### Culture Sources and Associated Infection Types

For most CRE and ESBL-E cases, including those in children <1 year of age, organisms were isolated from urine (131 [82.4%] from CRE cases and 196 [94.7%] from ESBL-E cases) (Table 4). Accordingly, the most common reported infection type was lower urinary tract infection (89 [56.3%] for CRE and 125 [74.0%] for ESBL-E). For CRE and ESBL-E cases, the greatest number of isolates were collected in an emergency department or outpatient setting (108 [68.4%] for CRE and 154 [91.1%] for ESBL-E), although CRE cases were more likely than ESBL-E cases to be hospital onset (40 [25.3%] vs. 13 [7.7%]; p<0.01) (Table 3). Healthcare-associated community onset (68 [43.0%] CRE vs. 40 [23.7%] ESBL-E) and community-associated (43 [27.2%] CRE vs. 109 [64.5%] ESBL-E) represented most CRE and ESBL-E cases.

**Table 4:** Isolate culture source, collection location, and infection types among incident pediatric CRE and ESBL-E cases, United States*

Category	CRE, 2016–2020	ESBL-E, 2019–2020†	p value
Culture source	n = 159	n = 207	
Urine‡	131 (82.4)	196 (94.7)	**<0.01**
Blood	20 (12.6)	8 (3.9)	**<0.01**
Other normally sterile site	8 (5.0)	3 (1.5)	**0.05**
Isolate collection location§	n = 158	n = 169	
Acute care hospital	49 (31.0)	15 (8.9)	**<0.01**
Outside acute care hospital	108 (68.4)	154 (91.1)	**<0.01**
Emergency department	16/108 (14.8)	0	**<0.01**
Outpatient setting	92/108 (85.2)	154/154 (100.0)	**<0.01**
Long-term care facility	0	0	NA
Long-term acute care facility	0	0	NA
Unknown	1 (0.6)	0	0.48
Infection types¶			
Lower urinary tract infection#	89 (56.3)	125 (74.0)	**<0.01**
Pyelonephritis	9 (5.7)	9 (5.3)	0.88
Bacteremia**	20 (12.7)	9 (5.3)	**0.02**
Other infection types	27 (17.1)	14 (8.3)	**0.02**
None	21 (13.3)	19 (11.2)	0.57
Unknown	9 (5.7)	8 (4.7)	0.70
Healthcare exposures in prior year			
Acute care hospitalization	74 (46.8)	38 (22.5)	**<0.01**
Resident of long-term care facility	0	2 (1.2)	0.27
Admission to long-term acute care hospital	0	0	NA
Inpatient or outpatient surgery††	61 (38.6)	16 (9.5)	**<0.01**
Chronic dialysis	5 (3.2)	0	**0.03**
Indwelling device in the 2 d before DISC			
Urinary catheter	29 (18.4)	20 (11.8)	0.10
Central venous catheter	37 (23.4)	12 (7.1)	**<0.01**
Any other device	60 (38.0)	19 (11.2)	**<0.01**
IV or oral antimicrobial use 30 d before DISC‡‡	N/A	52 (30.8)	N/A
None of the above healthcare exposures	43 (27.2)	110 (65.1)	**<0.01**
Outcomes, no. patients			
Hospitalization among community-onset cases§§	40/111 (36.0)	13/149 (8.7)	**<0.01**
ICU admission ≤6 d after DISC	10 (6.3)	5 (3.0)	0.15
30-d mortality			
Cases with an incident blood or sterile site specimen	4/27 (14.8)	2/9 (22.2)	0.29
Cases with an incident urine specimen	2/131 (1.5)	0	0.23

*Values are no. (%) except as indicated. Denominators are indicated when different from total number. Fisher exact test used for comparative statistics when >20% of expected cell counts were <5. Boldface indicates p<0.05. CRE, carbapenem-resistant Enterobacterales; DISC, date of incident specimen collection; ESBL-E, extended-spectrum β-lactamase–producing Enterobacterales; IV, intravenous. †ESBL-E surveillance was completed for 6 mo during 2019. ‡Two incident urine CRE cases with subsequent nonincident blood cultures were identified. One incident urine ESBL-E case with subsequent nonincident blood culture was identified. Nonincident blood cultures are not counted here. §Isolate collection location, epidemiologic class, infection types available for cases with completed case report forms only. ¶Cases could have >1 type of associated infection reported. #Lower urinary tract infection includes cases involving infection of the bladder or urethra. Pyelonephritis, or infections involving the kidney(s), were counted separately. **Bacteremia includes cases with a positive blood specimen or a documented diagnosis of sepsis, septicemia, bacteremia, or blood stream infection. 3 CRE cases of sepsis and 2 ESBL-E cases of sepsis (with or without blood cultures) are included in the bacteremia classification above. ††Surgery defined as procedures occurring in an operating room where a surgeon makes >1 incision through skin or mucous membrane, including laparoscopic approach. Ambulatory surgery centers may be included. ‡‡Antimicrobial use (intravenous or oral) in 30 d before DISC collected for ESBL-E cases only. §§Hospitalization at time of or within 30 d after DISC.

### Healthcare Exposures and Outcomes

Among cases with available case report form data, a greater proportion of children with CRE than ESBL-E underwent acute care hospitalization (74 [46.8%] CRE vs 38 [22.5%] ESBL-E; p<0.01) or surgery (61 [38.6%] CRE vs. 16 [9.5%] ESBL-E; p<0.01) within 1 year before specimen collection (Table 4). In the 2 days before specimen collection, CRE cases were also more likely than ESBL-E cases to have a central venous catheter (37 [23.4%] CRE vs. 12 [7.1%] ESBL-E; p<0.01) or other indwelling device (excluding urinary catheter) (60 [38.0%] CRE vs. 19 [11.2%] ESBL-E; p<0.01). ESBL-E cases were significantly more likely than CRE cases to have no reported healthcare exposures (110 [65.1%] vs. 43 [27.2%]; p<0.01). Among ESBL-E cases, antimicrobial use was documented in the 30 days before date of incident specimen collection for 52 (31.8%).

Hospitalization at the time of or within 30 days of specimen collection was required for a greater proportion of community-onset CRE (40 [36.0%]) versus ESBL-E (13 [8.7%]) cases (p<0.01). Median duration of admission among all hospitalized community-onset and hospital-onset cases was 18 days (IQR 3–103 days) for those with CRE versus 10 days (IQR 4–43 days) for those with ESBL-E (p = 0.34).

### Isolate Testing

The antimicrobial resistance profiles of incident CRE and ESBL-E cases from local clinical laboratories are shown elsewhere (Appendix Table 1). Among the 86 CRE isolates submitted for carbapenemase testing, 9 (10.5%) isolates from 6 of the 10 EIP sites harbored a carbapenemase: 4 *bla*_NDM_, 3 *bla*_OXA-48_-like, and 2 *bla*_KPC_ (Table 1). Distribution of CP-CRE varied by organism. The 9 CP-CRE isolates were from 9 children, fewer than half of whom were 1–3 years of age (3 [15.8%]) and 4–9 years of age (3 [13.0%]); 6 (18.8%) were from children with no reported underlying conditions (Appendix Table 2). The most common source was urine (8 CP-CRE isolates, 11.6% of submitted). Among the 11 CRE isolates from 2016–2018 that were sequenced, identified multilocus sequence types (STs) were diverse (Appendix Table 3); 2 of 11 isolates harbored CP genes. Separately, of the 7 ESBL-E organisms that underwent whole-genome sequencing at CDC, 6 were *E. coli*; ST131 and potential acquired ESBL gene *bla*_CTX-M-15_ were most common (Appendix Table 4).

## Discussion

Over a 5-year surveillance period, 159 incident pediatric CRE cases were reported across 10 EIP sites (representing >4 million children), resulting in an overall crude incidence of 0.70 cases/100,000 pediatric population. The CRE case estimate is lower than the 207 incident pediatric ESBL-E cases identified over 1.5 years across 6 EIP sites (>600,000 children), which corresponds to an average crude incidence of 23.08 cases/100,000 pediatric population. The burden of infections was higher among girls than boys, more were detected in urine than in sterile site cultures, and incidence was disproportionately high among children <1 year of age. We found variation in rates of infections by year, geographic region, and species and in the percentages of CRE organisms that produced carbapenemases.

Similar to findings of other studies (*17*,*30*), in our study, *E. coli* accounted for many resistant isolates and represented most ESBL-E species identified. Of note, *E. cloacae* complex comprised most of the incident CRE cases. Our finding differs from that of another large, nationally representative pediatric study conducted during 1999–2012 (*30*), in which the most common organisms identified were *E. coli* and *Proteus mirabilis*. It also differs from that of an earlier EIP study of adult CRE cases conducted during 2012–2013, in which *K. pneumoniae* accounted for most CRE cases identified (*5*). The previous EIP study used a different case definition that was more specific for carbapenemase-producing CRE (i.e., excluded ertapenem and required carbapenem nonsusceptibility and third-generation cephalosporin resistance) and thus may have excluded certain species that are less likely to produce carbapenemases, probably affecting the comparison to the cases in our study (*31*). From our sample of 47 pediatric CR–*E. cloacae* complex isolates submitted for carbapenemase testing, only 1 was confirmed to be a carbapenemase producer (*bla*_KPC_). Thus, the variability in species predominance and limited number of carbapenemase genes identified in our study may result from differences in case definition.

Most CRE and ESBL-E cases in our study were healthcare-associated infections with community onset or were community-associated infections. Strikingly, nearly 65% of the ESBL-E cases were reported to be community associated, and patients had no reported history of healthcare exposure. In addition, most cases of CRE and ESBL-E were detected from cultures collected outside an acute care hospital, a finding that differs from previous studies reporting pediatric CRE infections more commonly isolated from hospitalized patients (*30*). The most common source of CRE and ESBL-E in this study was urine (and lower urinary tract infections), which probably contributed to the high proportion of cases collected in outpatient settings. Increasing prevalence of community-associated ESBL-E urinary tract infections has been noted across patients of all ages (*32*,*33*). Our findings highlight a similar shift in the clinical epidemiology of multidrug-resistant infections in children, supported by a rising trend in community-acquired ESBL-E causing urinary tract infections in children (*34*). Continued implementation of national programs to detect, prevent, and treat multidrug-resistant infections must increasingly include pediatric populations and outpatient settings.

Although incidence of CRE was lower than that of ESBL-E, children with CRE infection were generally hospitalized for longer durations, and rates of intensive care unit admission were higher. Children with CRE infection were also more likely to have >1 underlying condition and prior healthcare exposure. Meropol et al. (*19*) and Logan et al. (*30*) also reported higher proportions of underlying conditions in children with multidrug-resistant gram-negative infections. However, our direct comparison of CRE and ESBL-E epidemiology revealed statistically significant clinical differences even among children with multidrug-resistant gram-negative infections. We also observed the proportion of CRE and ESBL-E in patients with no underlying conditions to be markedly higher than that found in previous studies focused on adult populations with those infections (*5*,*9*).

CRE incidence rates fluctuated by region throughout the study period, partly reflecting variation in the small number of cases occurring on a yearly basis. Separately, the overall incidence of ESBL-E clearly decreased during 2020. That finding contrasts with reports of increased rates of ESBL-E infections among hospitalized patients, primarily adults, in 2020 (*2*). Our data were unadjusted and included nonhospitalized patients, and it is possible that declines in outpatient healthcare use during the pandemic may have affected rates of ESBL-E among children. In addition, our data represent only mid-2019 through 2020, making the decline more difficult to interpret.

We also found annual CRE and ESBL-E incidence rates to be higher for female children and infants (most >1 month of age) compared with other age groups. We suspect that rates of antimicrobial-resistant infections were higher among girls in part because of increased testing in those populations resulting from a higher number of baseline urinary tract infections. The epidemiology among infants may differ from that among the overall pediatric population because of risk factors associated with infection acquired in neonatal intensive care units (*35*–*37*), vertical transmission (*38*,*39*), and higher rates of fecal colonization with antimicrobial-resistant *Enterobacteriaceae* (*40*). Recent evidence highlights how the human microbiome undergoes marked developmental progression over the first 2 years of life (*41*). Consistent with such a maturation process, Darda et al. observed spontaneous decolonization within 12 months among all neonates colonized with carbapenem-resistant gram-negative bacteria (*42*). Nonetheless, even when limited to prenatal and intrapartum exposures, antimicrobial drugs can profoundly affect the infant microbiome, involving expansion of gram-negative populations (proteobacteria) and supporting our observation of differences in the epidemiology among infants (*43*). A more focused look at that age group may be noteworthy for future studies.

Among the several limitations inherent in the use of surveillance systems, the case definitions for CRE and ESBL-E relied on susceptibility testing performed locally, and methods varied across laboratories. In addition, automated testing instruments at clinical laboratories may be more likely than other test methods to overdiagnose CRE. Second, data were retrospectively abstracted from medical records, and the quality of medical record documentation can vary between healthcare system and facility types, resulting in differences in reporting for some data elements. In addition, medical records were reviewed for the child only, and no maternal information was included in chart review, which may have limited identification of household risk factors (e.g., international travel by family, previously established as a risk factor for CRE and ESBL-E). Separately, because ≈97% of the incident ESBL-E cases from 1 site in 2020 did not have a case report form completed, no data beyond the clinical laboratory report were available. Third, isolate collection represents a convenience sample and may not be representative of all cases. In addition, we have limited data on STs because not all isolates submitted to CDC were sequenced. Fourth, we acknowledge that data for ESBL-E in our study are limited to 1.5 years and collected from fewer sites than surveillance data for CRE. Last, although the surveillance system includes geographically diverse catchment areas, it is not designed to be representative of the entire US pediatric population.

In summary, we found that CRE infections occurred less frequently than ESBL-E infections among US children but were more often associated with healthcare risk factors and hospitalization. Despite annual and geographic variation in the incidence of CRE and ESBL-E, the rate of infection for both pathogens was consistently highest among infants. Our descriptive data about major antimicrobial-resistant pathogens among children support continued infection prevention and control practices and antimicrobial stewardship in pediatric healthcare settings, particularly for patients in the youngest age group.

Appendix Additional information for study of carbapenem-resistant and extended-spectrum β-lactamase–producing Enterobacterales among children, United States, 2016–2020.
