# Supplementary material for: Carbapenem-Resistant and Extended-Spectrum β-Lactamase–Producing Enterobacterales in Children, United States, 2016–2020
Source: Emerg Infect Dis. 2024 Jun;30(6):1104–14. doi: 10.3201/eid3006.231734 (PMC11138972; doi:10.3201/eid3006.231734)
Supplement: Appendix — Additional information for study of carbapenem-resistant and extended-spectrum β-lactamase–producing Enterobacterales among children, United States, 2016–2020. [file 23-1734-Techapp-s1.pdf]

Article DOI: https://doi.org/10.3201/eid3006.231734

EID cannot ensure accessibility for supplementary materials supplied by authors. Readers who have difficulty accessing supplementary content should contact the authors for assistance.

Carbapenem-Resistant and Extended-Spectrum β-Lactamase–Producing Enterobacterales in Children, United States, 2016–2020

Appendix

Supplemental Methods

Total Surveillance Population

The total population of the 10 participating areas for CRE surveillance in 2020 was an estimated 23.2 million; this included Atlanta, Georgia area (8 counties, estimated population 4,202,188), Minneapolis/St. Paul, Minnesota (2 counties, estimated population 1,833,917), Portland, Oregon (3 counties, estimated population 1,837,201), Denver, Colorado (5 counties, estimated population 2,831,052), Baltimore, Maryland (4 counties, estimated population 1,945,451), Albuquerque, New Mexico (1 county, estimated population 676,444), Rochester, New York (1 county, estimated population 759,443), Nashville, Tennessee (8 counties, estimated population 1,817,304), San Francisco, California (3 counties, estimated population 3,722,245), and all of Connecticut (8 counties, estimated population 3,605,944).

The total population of the 6 participating areas for ESBL-producing Enterobacterales surveillance in 2020 was an estimated 3.0 million; this included counties of Atlanta, Georgia (2 counties, estimated population 441,832), Boulder, Colorado (1 county, estimated population 330,758), Baltimore Maryland (1 county, estimated population 585,708), Albuquerque, New Mexico (1 county, estimated population 676,444), Rochester, New York (1 county, estimated population 759,443), and middle Tennessee (4 counties, estimated population 164,106).

Normally Sterile Body Sites

For this surveillance, normally sterile sites included blood, cerebrospinal fluid, pleural fluid, pericardial fluid, peritoneal fluid, synovial fluid, bone, internal body tissue (lymph node, brain, heart, liver spleen, vitreous fluid, kidney, pancreas, or ovary), muscle, deep tissue, or samples from other normally sterile sites.

Data Collection

Both CRE and ESBL-E cases were identified through a query of automated testing instruments based on the protocols of the laboratories. Antimicrobial susceptibility test methods varied among the clinical laboratories although the majority reported the use of an automated test system (MicroScan, Beckman Coulter Diagnostics, Brea, CA; Vitek, bioMérieux, Marcy-l'Étoile, FR, or BD Phoenix, Becton Dickinson, Franklin Lakes, NJ). Kirby Bauer and E-tests were often used for confirmatory testing.

For cases that underwent a medical record review, one of the data elements collected included hospitalization in the year preceding the date of incident specimen collection. For cases ≤12 months of age, if the birth hospitalization included a neonatal intensive care unit (NICU) stay with discharge, or there were any additional hospitalizations after the birth hospitalization with discharge, then the infant would be considered to have had a prior hospitalization. If the birth hospitalization was otherwise in a well-newborn nursery or that was the only hospitalization prior to the initial invasive CRE or ESBL-E culture, then that would not count as a prior hospitalization. If the infant was in the NICU and had never been discharged home, this would also not count as a prior hospitalization.

Isolate Collection

A convenience sample of isolates from all EIP sites was submitted to CDC for confirmatory and molecular characterization. Sampling approach was determined separately by each of the participating clinical laboratories based on staff time and resources available for isolate selection and storage. Laboratories typically chose isolates meeting the case definition which were readily available and accessible for shipment to CDC.

Whole Genome Sequencing

Whole genome sequencing was conducted on a subset of CRE isolates that were confirmed carbapenem resistant using reference broth microdilution at CDC. CRE sequencing

criteria changed slightly each year to reduce the overall volume and burden on sequencing labs. For 2016 isolates, CDC sequenced all isolates that confirmed as CRE after antimicrobial susceptibility testing. For 2017 isolates, CDC sequenced isolates that confirmed as CRE after antimicrobial susceptibility testing, but excluded Ertapenem-monoresistant *E. cloacae* complex and *K. aerogenes.* For 2018 isolates, CDC sequenced isolates that confirmed as CRE after antimicrobial susceptibility testing, but excluded Ertapenem-monoresistant *E. cloacae* complex.

Whole genome sequencing was performed on all ESBL-E isolates received at CDC. Sequencing was performed using an Illumina MiSeq or NovSeq system (San Diego, CA). For MiSeq, genomic DNA was extracted using the Promega Maxwell 16 Low Elution Volume DNA Purification Kit and the Maxwell 16 MDx Instrument (Madison, WI, United States). For NovSeq, genomic DNA was sheared to a mean size of 600 bp using a Covaris LE220 focused ultrasonicator (Covaris Inc., Woburn, MA). DNA fragments were Ampure (Beckman Coulter Inc., Indianapolis, IN) cleaned and used to prepare dual-indexed sequencing libraries using NEBNext Ultra library prep reagents (New England Biolabs Inc., Ipswich, MA) and barcoding indices synthesized in the CDC Biotechnology Core Facility. Libraries were analyzed for size and concentration, normalized, pooled and denatured for loading onto flowcells for cluster generation. Sequencing was performed on a Novaseq using Novaseq 2x250bp paired-end sequencing kits. On completion, sequence reads were filtered for read quality, basecalled and demultiplexed using bcl2fastq (v2.19). All sequences were analyzed using the CDC laboratory's in-house QuAISAR-H pipeline (Quality, Assembly, species Identification, Sequence typing, Annotation, Resistance mechanisms for Hospital acquired infections; https://github.com/DHQP/QuAISAR_singularity). QuAISAR-H identified antibiotic resistant (AR) genes, including carbapenemase genes, beta-lactamase genes, and plasmid-mediated colistin resistance (*mcr*) genes, using a non-redundant combined database of acquired AR genes from ResFinder; ARG-ANNOT, and AMRFinderPlus; genes with a minimum of 98% identity and 90% coverage threshold were reported. Multilocus sequence type (MLST) was determined using the publicly available schemes curated by pubMLST (last accessed 07/15/2022).

Appendix Table 1. Antimicrobial resistance of incident carbapenem-resistant Enterobacteriaceae (CRE) and extended-spectrum beta-lactamase-producing Enterobacterales (ESBL-E) cases by organism based on testing by local clinical laboratories

Antimicrobial Agent	CRE (2016-2020) No. resistant / no. tested (%)	ESBL (2019-2020) No. resistant / no. tested (%)
Aminoglycosides	29/151 (19.2%)	44/157 (28.0%)
Amikacin	6/115 (5.2%)	3/99 (3.0%)
Gentamicin	23/150 (15.3%)	38/156 (24.4%)
Tobramycin	25/142 (17.6%)	39/125 (31.2%)
Carbapenems	153/154 (99.4%) *	0/161 (0%)
Doripenem	0/21 (0%)	0/8 (0%)
Ertapenem	141/149 (94.6%) *	0/169 (0%)
Imipenem	28/95 (29.5%)	0/95 (0%)
Meropenem	22/114 (19.3%)	0/107 (0%)
Extended-spectrum cephalosporins	128/148 (86.5%)	164/164 (100%)
Cefotaxime	30/46 (65.2%)	26/26 (100%)
Ceftazidime	79/103 (76.7%)	82/111 (73.9%)
Ceftriaxone	122/142 (85.9%)	162/163 (99.4%)
Cefepime	53/140 (37.9%)	80/150 (53.3%)
Cefoxtin[b]	---	---
Fluoroquinolones	45/150 (30.0%)	79/147 (53.7%)
Ciprofloxacin	43/142 (30.3%)	73/139 (52.5%)
Levofloxacin	24/109 (22.0%)	49/107 (45.8%)
Beta-lactam and non-beta-lactam combination agents		
Amoxicillin-clavulanate	29/79 (36.7%)	28/76 (36.8%)
Piperacillin-tazobactam	96/138 (69.6%)	14/151 (9.3%)
Ceftazidime-avibactam	0/12 (0%)	0/2 (0%)
Meropenem-vaborbactam	0/3 (0%)	0/2 (0%)
Imipenem-relebactam *	---	---
Folate pathway antagonists		
Trimethoprim-sulfamethoxazole	29/148 (19.6%)	64/160 (40.0%)
Other antimicrobials		
Aztreonam	64/95 (67.4%)	69/90 (76.7%)
Colistin †	1/3 (33.3%)	---
Fosfomycin	0/2 (0%)	0/17 (0%)
Nitrofurantoin	14/134 (10.5%)	5/152 (3.3%)
Tigecycline	0/42 (0%)	0/15 (0%)

CRE: Carbapenem-Resistant Enterobacterales; ESBL-E: Extended-Spectrum β-Lactamase-Producing Enterobacterales.
*A minimum inhibitory concentration (MIC) >1 for ertapenem was considered sufficient for meeting the phenotypic case definition for MuGSI CRE surveillance. This table reports cases based on MIC at the local clinical laboratories, not the reported interpretation. One clinical laboratory in this study uses an automated testing instrument (ATI) card with the highest MIC for ertapenem being 1. This result was reported as R to clinicians while awaiting confirmatory testing, but does not meet the MIC > 1 breakpoint used in this table.
†No CRE or ESBL-E isolates were tested for cefoxitin or imipenem-relebactam susceptibility and no ESBL-E isolates were tested for colistin susceptibility.

Appendix Table 2. Characteristics of pediatric carbapenemase-producing Enterobacterales (CP-CRE) Cases, 2016–2020

Characteristic	Total Cases	Isolates Submitted for Carbapenemase Testing	No. of Carbapenemase-Producing Isolates (%) *
Year of Specimen Collection			
2016	31	8	0
2017	20	10	0
2018	34	22	2 (9.1)
2019	43	25	6 (24.0)
2020	31	21	1 (4.8)
Age Category			
< 1 y	35	22	1 (4.6)
1–3 y	30	19	3 (15.8)
4–9 y	47	23	3 (13.0)
10–14 y	28	11	2 (18.2)
15–17 y	19	11	0
Gender			
Female	94	46	5 (10.9)
Male	64	40	4 (10.0)
Unknown	1	0	0
Race			
White	79	44	4 (9.1)
Black	29	20	1 (5.0)
Other/Unknown	51	22	4 (18.2)
Ethnicity			
Hispanic	43	16	2 (12.5)

Characteristic	Total Cases	Isolates Submitted for Carbapenemase Testing	No. of Carbapenemase-Producing Isolates (%) *
Non-Hispanic	86	54	7 (13.0)
Unknown	30	16	0
Source			
Blood	20	13	1 (7.7)
Other Sterile	8	4	0
Urine	131	69	8 (11.6)
Collection Location			
Acute Care Hospital	50	32	2 (6.25)
Outpatient setting or ER	108	54	7 (13.0)
Unknown	1	0	0
Any Underlying Conditions			
0	59	32	6 (18.8)
≥1	99	54	3 (5.6)
Unknown	1	0	0
Epidemiologic Classification			
Hospital Onset	41	28	2 (7.1)
Community Associated	43	24	4 (16.7)
Healthcare Associated Community Onset	68	34	3 (8.8)
Unknown	7	0	0
International Travel			
Yes	6	4	2 (50.0)
No / Unknown	153	82	7 (8.5)

CRE: Carbapenem-Resistant Enterobacterales; ESBL-E: Extended-Spectrum β-Lactamase-Producing Enterobacterales; ER: Emergency Room.
* Percent shown is of percent total isolates submitted to CDC for testing.

Appendix Table 3. Multilocus sequence types, carbapenemase, and beta-lactamase genes from pediatric carbapenem-resistant Enterobacteriaceae (CRE) isolates that underwent whole genome sequencing (n=11)

Organism	Multilocus sequence types	Carbapenemase genes	Non-carbapenemase beta-lactamase genes
Enterobacter cloacae complex *(n=5)*	ST133(Pasteur)	NA	$\textit{bla}_{\text{ACT-86}}$ *
	ST252(Pasteur)	NA	$\textit{bla}_{\text{ACT-3}}$
	ST467(Pasteur)	NA	$\textit{bla}_{\text{ACT-17}}$
	ST50(Pasteur)	NA	$\textit{bla}_{\text{ACT-15}}$
	ST526(Pasteur)	NA	$\textit{bla}_{\text{MIR-16}}$
Escherichia coli (n=3)	ST1123(Pasteur), ST11538(Achtman)	NA	$\textit{bla}_{\textit{AmpC1}}$, $\textit{bla}_{\textit{AmpH}}$, $\textit{bla}_{\text{CMY-2}}$, $\textit{bla}_{\text{EC-8}}$ †
	ST963(Pasteur), ST963(Achtman)	NA	$\textit{bla}_{\textit{AmpC1}}$, $\textit{bla}_{\textit{AmpH}}$, $\textit{bla}_{\text{CMY-2}}$, $\textit{bla}_{\text{EC-8}}$ †
	ND (Pasteur), ST13455(Achtman),	$\textit{bla}_{\text{OXA-48-like}}$	$\textit{bla}_{\textit{AmpH}}$, $\textit{bla}_{\text{CTX-M-15}}$, $\textit{bla}_{\text{EC-19}}$**, $\textit{bla}_{\text{SHV-12}}$
Klebsiella aerogenes (n=2)	ST205(Pasteur)	NA	$\textit{bla}_{\text{CMY2-MIR-ACT-EC}}$ ‡
	ST373(Pasteur)	$\textit{bla}_{\text{NDM-1}}$	$\textit{bla}_{\text{CTX-M-15}}$, $\textit{bla}_{\text{TEM-1a}}$, $\textit{bla}_{\text{CMY2-MIR-ACT-EC}}$ §
Klebsiella pneumoniae (n=1)	ST1426(Pasteur)	NA	$\textit{bla}_{\text{AmpH}}$, $\textit{bla}_{\text{CTX-M-15}}$, $\textit{bla}_{\text{OXA-1}}$, $\textit{bla}_{\text{SHV-11}}$, $\textit{bla}_{\text{TEM1}}$

ESBL-E: Extended-spectrum beta-lactamase-producing Enterobacterales; ST: Sequence Type; ND: Not Determined.
* This call is a mutant/novel allele that has 5 amino acid changes from the listed gene.
† This call is a mutant/novel allele that has 7 amino acids different from the listed gene.
‡ This call is a mutant/novel allele that is 1 amino acid different from the listed gene.
§ This call is a mutant/novel allele that has 3 amino acids different from the listed gene.

Appendix Table 4. Multilocus sequence types, acquired extended-spectrum beta-lactamases (ESBL), and beta-lactamase genes identified from pediatric ESBL-E isolates that underwent whole genome sequencing (n=7)

Organism	Sequence Type (scheme)	Acquired ESBL genes	beta-lactamase genes
Escherichia coli (n=6)	ST43(Pasteur), ST131(Achtman)	$bla_{\text{CTX-M-15}}$	bla_{AmpH}, $bla_{\text{EC-5}}$, $bla_{\text{OXA-1}}$
	ST43(Pasteur), ST131(Achtman)	$bla_{\text{CTX-M-27}}$	bla_{AmpH}, $bla_{\text{EC-5}}$
	ST506(Pasteur), ST131(Achtman)	$bla_{\text{CTX-M-15}}$	bla_{AmpH}, $bla_{\text{EC-5}}$, $bla_{\text{TEM-1}}$
	ST2(Pasteur), ST167(Achtman)	$bla_{\text{CTX-M-55}}$	bla_{AmpC1}, bla_{AmpH}, $bla_{\text{EC-15}}$
	ST87(Pasteur), ST58(Achtman)	$bla_{\text{CTX-M-15}}$	bla_{AmpC1}, bla_{AmpH}, $bla_{\text{EC-18}}$, $bla_{\text{CMY-2}}$
	ND(Pasteur), ST648(Achtman)	$bla_{\text{CTX-M-15}}$	bla_{AmpC1}, bla_{AmpH}, $bla_{\text{EC-19}}$
Klebsiella pneumoniae (n=1)	ST307(Pasteur)	$bla_{\text{CTX-M-15}}$	bla_{AmpH}, $bla_{\text{OXA-1}}$, $bla_{\text{SHV-28}}$, $bla_{\text{TEM-1}}$

ST: Sequence Type; ND: Not Determined.
